# Clinical characteristics of tumor lysis syndrome in childhood acute lymphoblastic leukemia

**DOI:** 10.1038/s41598-021-88912-2

**Published:** 2021-05-06

**Authors:** Yao Xue, Jing Chen, Siyuan Gao, Xiaowen Zhai, Ningling Wang, Ju Gao, Yu Lv, Mengmeng Yin, Yong Zhuang, Hui Zhang, Xiaofan Zhu, Xuedong Wu, Chi Kong Li, Shaoyan Hu, Changda Liang, Runming Jin, Hui Jiang, Minghua Yang, Lirong Sun, Kaili Pan, Jiaoyang Cai, Jingyan Tang, Xianmin Guan, Yongjun Fang

**Affiliations:** 1 Department of Hematology and Oncology, Children’s Hospital of Nanjing Medical University, Nanjing, China; 2 Key Laboratory of Hematology, Nanjing Medical University, No. 72 Guangzhou Road, Nanjing, 210008 China; 3 Department of Hematology/Oncology, Shanghai Children’s Medical Center, National Health Committee Key Laboratory of Pediatric Hematology & Oncology, School of Medicine, Shanghai Jiao Tong University, Shanghai, China; 4 Department of Hematology/Oncology, Ministry of Education Key Laboratory of Child Development and Disorders, National Clinical Research Center for Child Health and Disorders, China International Science and Technology Cooperation Base of Child Development and Critical Disorders, Chongqing Key Laboratory of Pediatrics, Children’s Hospital of Chongqing Medical University, Chongqing, China; 5 Hematology Department, Children’s Hospital of Fudan University, Shanghai, China; 6 Department of Pediatric, The Second Hospital of Anhui Medical University, Hefei, China; 7 Department of Pediatrics, West China Second University Hospital, Sichuan University, Key Laboratory of Birth Defects and Related Disease of Women and Children, Ministry of Education, Chengdu, China; 8 Department of Hematology, Kunming Children’s Hospital, Kunming, China; 9 Department of Pediatric Hematology, Tongji Hospital of Tongji Medical College, Huazhong University of Science and Technology, Wuhan, China; 10 Department of Pediatrics, Qilu Hospital of Shandong University, Jinan, China; 11 Department of Hematology and Oncology, Guangzhou Women and Children’s Medical Center, Guangzhou, China; 12 State Key Laboratory of Experimental Hematology and Division of Pediatric Blood Diseases Center, Institute of Hematology and Blood Diseases Hospital, Chinese Academy of Medical Sciences and Peking Union Medical College, Tianjin, China; 13 Department of Pediatrics, Nanfang Hospital, Southern Medical University, Guangzhou, China; 14 Department of Pediatrics, Hong Kong Children’s Hospital, The Chinese University of Hong Kong, Hong Kong, SAR China; 15 Department of Hematology/Oncology, Children’s Hospital of Soochow University, Suzhou, China; 16 Department of Hematology/Oncology, Jiangxi Provincial Children’s Hospital, Nanchang, China; 17 Department of Pediatrics, Union Hospital of Tongji Medical College, Huazhong University of Science and Technology, Wuhan, China; 18 Department of Hematology/Oncology, Shanghai Children’s Hospital, Shanghai Jiao Tong University, Shanghai, China; 19 Department of Pediatrics, Xiangya Hospital Central South University, Changsha, China; 20 Department of Pediatrics, Affiliated Hospital of Qingdao University, Qingdao, China; 21 Department of Hematology/Oncology, Northwest Women’s and Children’s Hospital, Xi’an, China

**Keywords:** Acute lymphocytic leukaemia, Leukaemia

## Abstract

Tumor lysis syndrome (TLS) is a common and fatal complication of childhood hematologic malignancies, especially acute lymphoblastic leukemia (ALL). The clinical features, therapeutic regimens, and outcomes of TLS have not been comprehensively analyzed in Chinese children with ALL. A total of 5537 children with ALL were recruited from the Chinese Children’s Cancer Group, including 79 diagnosed with TLS. The clinical characteristics, treatment regimens, and survival of TLS patients were analyzed. Age distribution of children with TLS was remarkably different from those without TLS. White blood cells (WBC) count ≥ 50 × 10^9^/L was associated with a higher risk of TLS [odds ratio (OR) = 2.6, 95% CI = 1.6–4.5]. The incidence of T-ALL in TLS children was significantly higher than that in non-TLS controls (OR = 4.7, 95% CI = 2.6–8.8). Hyperphosphatemia and hypocalcemia were more common in TLS children with hyperleukocytosis (OR = 2.6, 95% CI = 1.0–6.9 and OR = 5.4, 95% CI = 2.0–14.2, respectively). Significant differences in levels of potassium (*P* = 0.004), calcium (*P* < 0.001), phosphorus (*P* < 0.001) and uric acid (*P* < 0.001) were observed between groups of TLS patients with and without increased creatinine. Laboratory analysis showed that older age was associated with a higher level of creatinine. Calcium level was notably lower in males. WBC count, lactate dehydrogenase, and creatinine levels were significantly higher in T-ALL subgroup, whereas procalcitonin level was higher in B-ALL children. Older age, infant, a higher level of WBC and T-ALL were risk factors TLS occurrence. Hyperleukocytosis has an impact on the severity of TLS, while renal injury may be an important feature in the process of TLS.

## Introduction

Acute lymphoblastic leukemia (ALL) is a type of cancer that accounts for 25% of all pediatric malignancies having developed before age of 15^1,2^. About 20% of patients may confront treatment failure, mainly due to relapse, secondary tumor, chemotoxicity, or severe complications^3^. Among the complications, tumor lysis syndrome (TLS) is common and fatal, especially in newly diagnosed patients. A deep understanding of TLS may benefit the overall outcome of childhood ALL.

TLS refers to a spectrum of disorders resulting from the rapid release of intracellular substances from lysed cells^4^. It is a potentially fatal clinical condition. TLS is characterized by clinical findings of hyperuricemia, hyperkalemia, hyperphosphatemia, and hypocalcemia^5,6^. Rapid progression of TLS often exerts severe toxic effects on organs, leading to renal impairment, epilepsy, cardiac arrhythmias, pulmonary edema, and even death. Clinical observations suggest that TLS tends to arise from highly proliferative malignancies, such as Burkitt’s lymphoma and ALL, in which tumor burden is heavy, or in response to initial therapy^7^. Therefore, TLS is a great challenge in childhood ALL for clinical physicians.

Previous studies have reported the risk factors and management standards for TLS^8,9^. However, few comprehensive analyses have been carried out to illustrate the clinical features, therapeutic regimens, and outcomes of TLS in ALL children^10,11^. Here, we analyzed the clinical data of TLS in ALL children from the Chinese Children’s Cancer Group (CCCG). The present findings will help to get a better understanding of TLS.

## Subjects and methods

### Patient recruitment

All patients included in this retrospective study were diagnosed with childhood ALL according to their morphology, immunology, cytogenetic and molecular biology between January 2015 and September 2018. Patient recruitment was conducted in 20 major hospitals or medical centers registered in CCCG, across 10 provinces, 3 municipalities directly under the Central Government in mainland China and Hong Kong (as listed in Authors’ information Section). The last follow-up was made in December 2018. The ages of patients ranged from 0 to 18 years. Laboratory TLS (LTLS) was defined as the metabolic disturbance of hyperkalemia, hyperphosphatemia, hyperuricemia, and hypocalcemia, while clinical TLS as LTLS along with renal injury/cardiac arrhythmias/seizures^7^. Finally, 5537 childhood ALL patients were included in our present study. A total of 79 TLS cases were reported from 13 institutions (as shown in the Acknowledgments section), among which 35 were diagnosed as clinical TLS. The research protocol was approved by the Medical Ethics Committee of the 20 institutions participating in this study and informed consent was obtained from children’s parents. All methods were performed following relevant guidelines and regulations expounded in Policies of the Nature Research journals.

### Treatment protocol

All patients were treated according to CCCG-ALL 2015 protocol (ChiCTR-IPR-14005706). Chemotherapy regimens were described in previous studies^12,13^. After the establishment of ALL diagnosis, the patients received dexamethasone for 4 days, as upfront window therapy, followed by remission induction from day 5 to day 28 with prednisone, vincristine, daunorubicin, and PEG-asparaginase. Bone marrow cell morphology and flow cytometric MRD were used to assess treatment response at day 19 and day 46. The risk level of these cases was determined according to CCCG-ALL 2015 protocol. Basis of risk escalation included T-ALL, age < 1 or > 10 years, gene fusion, chromosome abnormality, high white blood cell count at diagnosis, CNS involvement, etc. TLS was treated with hydration with about 1/4–1/3 dextrose normal saline (2–3 L/m^2^/day), diuretic therapy, or allopurinol.

### Data collection

The clinical characteristics, including age, gender, immunophenotype, risk category, time of diagnosis, highest white blood cell (WBC) count and the most abnormal value of blood biochemical test, special findings of clinical manifestation, treatment regimens, and survival were collected from all 20 institutions.

### Statistical analyses

Statistical analyses were performed using PASW Statistics 18. Continuous variables with normal distribution were summarized by mean ± standard deviation and compared by using two-tailed Student’s t-test. Continuous variables with non-normal distribution were expressed as median ± interquartile range (25th–75th percentile) and analyzed by Nonparametric test. Categorical variables were described with the number of subjects and compared using the Chi-square test. Multivariable logistic analysis was used to assess the association of clinical characteristics with TLS, as measured by the estimated odds ratio (OR) with the 95% confidence interval (95% CI). We performed logistic regression analyses to detect variables significantly associated with the prognosis of TLS. *P* value < 0.05 was considered statistically significant.

### Ethics approval and consent to participate

The study was approved by the Ethics Committee of Children's Hospital of each participating institution. All the guardians of participants signed an informed consent for participation in this study.

### Consent for publication

All the authors have reviewed and approved this manuscript and consented to publish this paper.

## Results

### Basic information of study subjects

A total of 5537 patients were recruited from 13 Chinese hospitals in the CCCG group between 2015 to 2018. After their data were confirmed, 79 TLS patients were included in this retrospective study.

Children older than 10 years and younger than 1 year had a higher risk of TLS than ALL children of 1–10 years old (OR = 2.2, 95% CI = 1.3–3.8 for the older group and OR = 8.6, 95% CI = 3.0–22.2 for the younger group). Children with WBC count ≥ 50 × 10^9^/L were more susceptible to TLS (OR = 2.6, 95% CI = 1.6–4.5). In addition, the incidence of T-ALL in TLS patients was significantly higher than that in other patients (OR = 4.7, 95% CI = 2.6–8.8). Multivariable logistic analysis demonstrated no significant association between TLS risk and other clinical characteristics (i.e., gene fusion, chromosome karyotype, and treatment branch) (Table 1).

**Table 1:** General information of childhood ALL patients with and without TLS.

	Without TLS 5458 (N%)	With TLS 79 (N%)	OR (95% CI)^c^
**Age**			
1 to 10	4689 (99.0)	45 (1.0)	1.00
≥ 10	689 (96.2)	27 (3.8)	**2.2 (1.3**–**3.8)**
< 1	80 (92.0)	7 (8.0)	**8.6 (3.0**–**22.2)**
**Sex**			
Female	2232 (99.0)	23 (1.0)	1.00
Male	3226 (98.3)	56 (1.7)	1.5 (0.9–2.5)
**WBC**^**a**^** (× 10**^**9**^**/L)**			
< 50	4351 (99.2)	36 (0.8)	1.00
≥ 50	1107 (96.3)	43 (3.7)	**2.6 (1.6**–**4.5)**
**Immunophenotype**			
B	4979 (99.2)	42 (0.8)	1.00
T	479 (92.8)	37 (7.2)	**4.7 (2.6**–**8.8)**
**t(12;21)(*ETV6-RUNX1*)**			
Present	1067 (99.5)	5 (0.5)	1.00
Absent	4391 (98.3)	74 (1.7)	0.9 (0.3–3.0)
**t(1;19)(*TCF3-PBX1*)**			
Absent	5185 (98.6)	74 (1.4)	1.00
Present	273 (98.2)	5 (1.8)	0.6 (0.2–1.8)
**t(9;22)(*BCR-ABL1*)**			
Absent	5231 (98.6)	76 (1.4)	1.00
Present	227 (98.7)	3 (1.3)	1.4 (0.5–6.0)
**KMT2A rearrangement**			
Absent	5285 (98.6)	74 (1.4)	1.00
Present	173 (97.2)	5 (2.8)	1.3 (0.5–4.3)
**Chromosome**			
Hyperdiploidy > 50	789 (99.4)	5 (0.6)	1.00
Others^b^	4669 (98.4)	74 (1.6)	0.9 (0.4–2.7)
**Risk**			
LR	2885 (99.6)	11 (0.4)	1.00
IR/HR	2573 (97.4)	68 (2.6)	1.8 (0.7–4.4)

^a^Maximum number of WBC.

^b^Seven missing values of Chromosome karyotype were classified as “others”.

^c^Calculated by multivariable logistic regression analyses.

Bold values denote significant difference between groups.

In the subgroup analysis of TLS children divided according to WBC count (Table 2), the results showed a higher proportion of T-ALL children in the high WBC count group (≥ 50 × 10^9^/L) (OR = 7.3, 95% CI = 2.6–19.9). In addition, the incidences of hyperphosphatemia and hypocalcemia were significantly higher in TLS children with hyperleukocytosis. It also seemed that proportion of ALL children aged < 1 year was higher in the high WBC count group than in the low WBC count group, but the difference was insignificant.

**Table 2:** Distribution of TLS patients grouped by WBC count.

	WBC < 50 × 10^9^/L (n = 36, %)	WBC ≥ 50 × 10^9^/L (n = 43, %)	OR (95% CI)
**Age**			
≥ 1	97.2	86.0	1.00
< 1	2.8	14.0	5.7 (0.7–49.6)
**Sex**			
Female	33.3	25.6	1.00
Male	66.7	74.4	1.5 (0.5–3.9)
**Extramedullary infiltration**^**a**^			
No	91.7	83.7	1.00
Yes	8.3	16.3	2.1 (0.5–9.0)
**Immunophenotype**			
B	77.8	32.6	1.00
T	22.2	67.4	**7.3 (2.6–19.9)**
**Hyperkalemia**			
No	75.0	55.8	1.00
Yes	25.0	44.2	2.4 (0.9–6.2)
**Hyperphosphatemia**			
No	44.4	23.3	1.00
Yes	55.6	76.7	**2.6 (1.0–6.9)**
**Hyperuricemia**			
No	38.9	48.8	1.00
Yes	61.1	51.2	0.7 (0.3–1.6)
**Hypocalcemia**			
No	72.2	32.6	1.00
Yes	27.8	67.4	**5.4 (2.0–14.2)**

^a^The meaning of extramedullary infiltration was CNS/Testicular leukemia, mediastinal mass as well as enlarged spleen/liver.

Bold values denote significant difference between groups.

### Clinical features and treatment data

**Figure 1:**
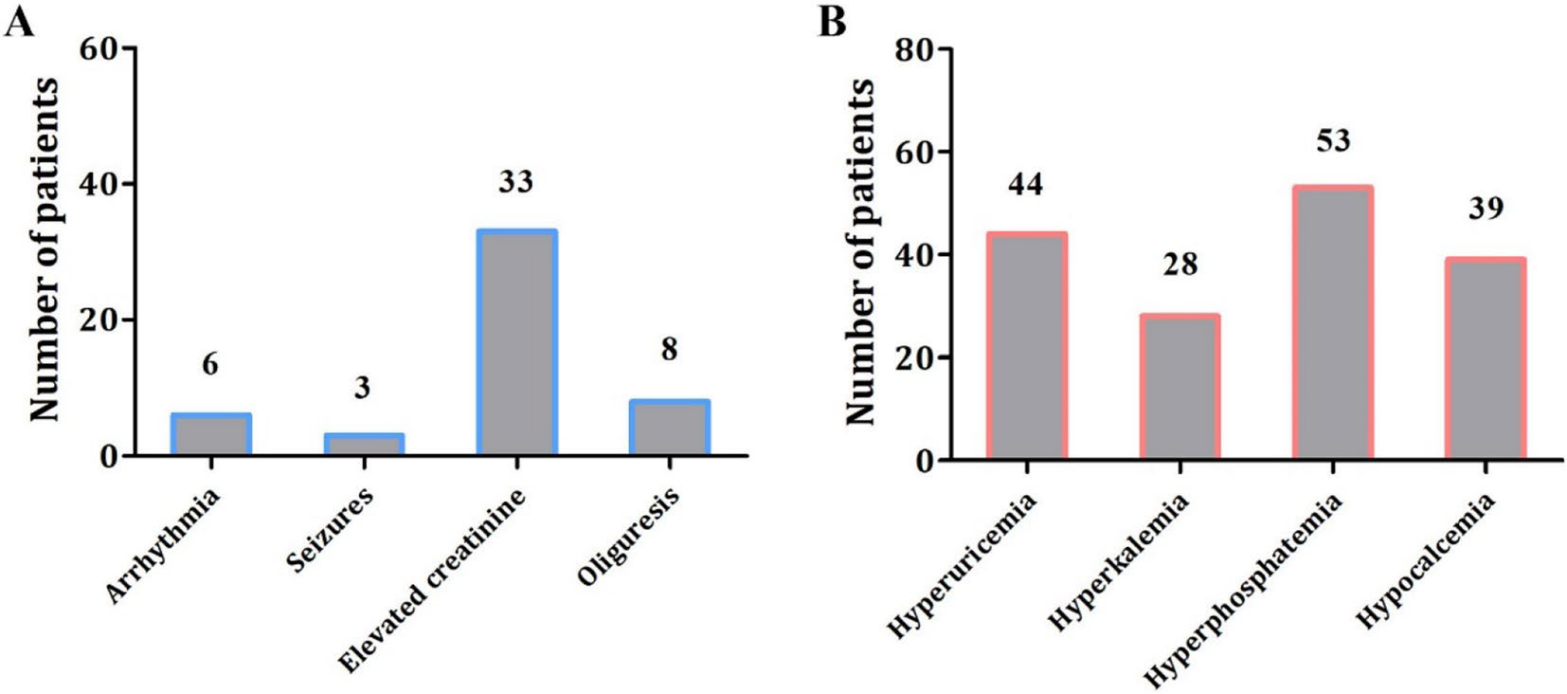
Distribution of clinical manifestation (**A**) and abnormal laboratory values (**B**) in TLS patients. It should be noted that the uric acid and phosphorus data were missing in 2 patients. Elevated creatinine: increased serum creatinine level of > 0.3 mg/dL (26.5 μmol/L) (or a single value > 1.5 times the upper limit of the age-appropriate normal range); oliguria: an average urine output of < 0.5 mL/kg/h for 6 h; hyperuricemia: uric acid > 8.0 mg/dL (475.8 μmol/L); hyperkalemia: potassium > 6.0 mmol/L; hyperphosphatemia: phosphorus > 6.5 mg/dL (2.1 mmol/L); hypocalcemia: corrected calcium < 7.0 mg/dL (1.75 mmol/L).

Clinical features were summarized in Fig. 1A. Among the 79 patients, arrhythmia was observed in 6 cases (7.5%), epilepsy in 3 cases (3.8%), high creatinine in 33 cases (41.7%), and oliguresis in 8 cases (10.1%). As to the distribution of abnormal laboratory indexes, hyperphosphatemia occurred with the highest frequency (68.8%) and hyperkalemia occurred with the lowest frequency (35.4%) (Fig. 1B). It should be noted that a few clinical data were missing.

After the initiation of the chemotherapy, TLS developed in 43 patients (54.4%) at 1–3 days, and in 28 patients (35.5%) at 4 days or later. Only 8 patients (10.1%) showed TLS before the chemotherapy. As shown in Supplementary Figure 1A–C, hydration lasted for less than 7 days in 28 patients, 7–13 days in 32 patients, and 14 days or even longer in another 17 patients. The median hydration treatment duration was 8 days (range 1–30 days). Diuresis was also applied in 60 TLS children for less than 7 days, and for a longer duration in the remaining 17 children, with a median of 3 days and a range of 0–15 days. In addition, allopurinol treatment was applied in 30 TLS children for 0–7 days, in 33 patients for 7–13 days, and in 14 patients for longer than 14 days, with a median of 8 days and a range of 0–30 days.

### Laboratory indexes

**Figure 2:**
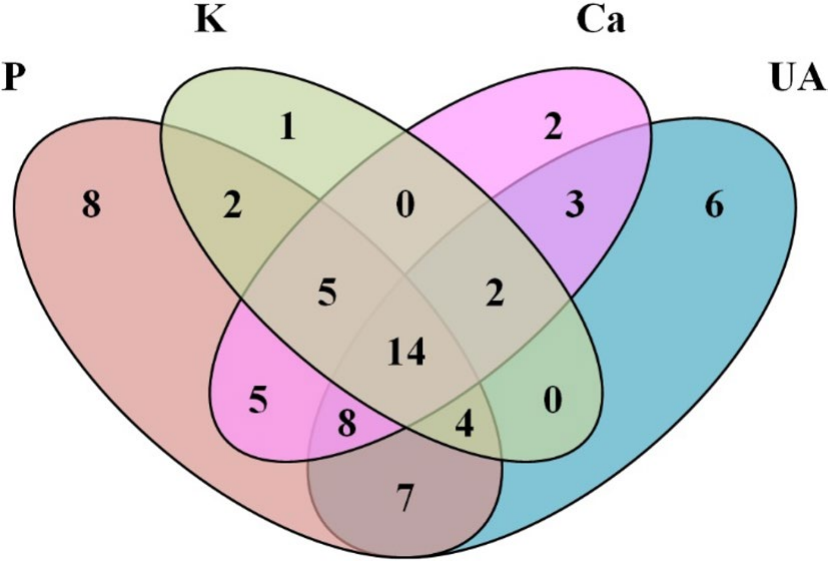
Venn diagram displaying the interrelationship among abnormal biochemical values. It should be noted that data of uric acid and phosphorus in 2 patients was missing. *K* potassium, *Ca* calcium, *P* phosphorus, *UA* uric acid.

The relationship among abnormal biochemical indexes is illustrated in the Venn diagram in Fig. 2. Hyperphosphatemia was found to be the most common abnormality, often accompanied by hyperkalemia (25/53) and frequently causing hypocalcemia (32/53). That is, only three patients (10.7%) with hyperkalemia and seven patients (17.9%) with hypocalcemia had normal levels of phosphorus. To identify features associated with clinical symptoms, we analyzed biochemical abnormalities among patients with/without arrhythmias, seizures, and renal injury (including increased creatinine level and oliguria). We found significant differences in levels of potassium, calcium, phosphorus, and uric acid between groups of TLS patients with or without high creatinine (Table 3). Besides, patients with oliguria had a higher level of potassium and a lower level of calcium.

**Table 3:** Difference of biochemical values among patients with/without clinical symptom of TLS.

	Arrhythmias		*P*	Seizures		*P*	Elevated creatinine		*P*	Oliguria		*P*
	Yes (6)	No (73)		Yes (3)	No (76)		Yes (33)	No (46)		Yes (8)	No (71)	
K (mmol/L)	4.5 ± 1.6	5.3 ± 1.3	0.173	6.1 ± 2.2	5.2 ± 1.3	0.259	5.7 ± 1.4	4.8 ± 1.1	**0.004**	6.5 ± 1.2	5.1 ± 1.3	**0.005**
Ca (mmol/L)	2.1 ± 0.6	1.8 ± 0.6	0.181	1.5 ± 0.8	1.8 ± 0.6	0.316	1.5 ± 0.6	2.0 ± 0.4	**< 0.001**	1.4 ± 0.6	1.8 ± 0.5	**0.036**
P^a^ (mmol/L)	1.7 (1.4–2.2)	3.3 (2.1–4.2)	**0.009**	–^b^	2.7 (1.9–4.2)	0.586	4.2 (3.2–5.2)	2.1 (1.6–2.7)	**< 0.001**	4.2 (2.2–4.3)	2.7 (1.9–4.2)	0.125
UA^a^ (umol/L)	358.4 (104.2–682.9)	644.8 (352.3–914.4)	0.116	–^b^	541.0 (344.2–874.0)	0.712	764.0 (495.0–1180.3)	414.0 (224.3–588.3)	**< 0.001**	785.9 (498.5–1192.0)	504.7 (326.5–821.1)	0.105

*K* potassium, *Ca* calcium, *P* phosphorus, *UA* uric acid.

^a^Represented by median (25–75 centile) because of non-normal distribution.

^b^Number of data was too small to be represented by 25–75 centile.

Bold values denote significant difference between groups.

Subsequently, all the 79 TLS patients were grouped by gender, age, immunophenotype, chromosome abnormality, gene fusion, and laboratory results. As shown in Supplementary Table 1, older age was associated with a higher level of creatinine. In addition, calcium level was remarkably lower in male. Subsequently, we found that WBC count, lactate dehydrogenase (LDH), and creatinine levels were significantly higher in T-ALL subgroup, while the procalcitonin (PCT) level was higher in B-ALL children (Supplementary Table 2).

### Outcomes

All the 79 patients received CCCG-2015 chemotherapy regimens after the diagnosis of ALL. Only one case lost to follow-up. Among the others, 4 cases died within 1–2 months after the diagnosis of TLS; 3 cases experienced a recurrence of ALL (within half a year in 1 case relapsed, after 1–2 years in 2 cases). The remaining of TLS children were cured and are surviving event-free at the time of writing (Month/Date/Year).

**Figure 3:**
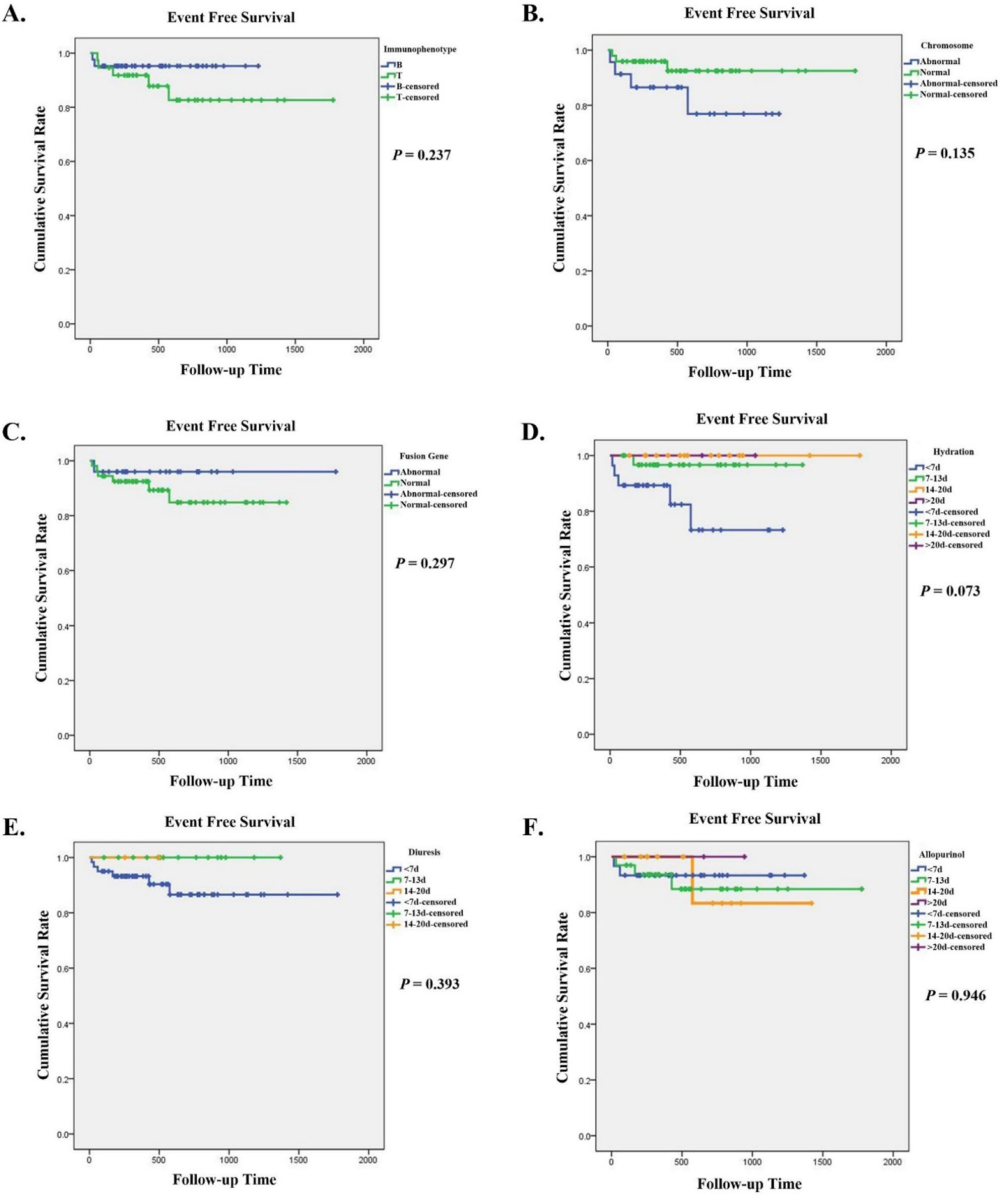
Role of immunophenotype (**A**), chromosome karyotype (**B**), fusion genes (**C**), and duration of treatment (**D**–**F**) in the prognosis of TLS patients.

The 78 children with complete follow-up data were grouped by their survival status. No statistically significant association was observed between important lab indexes and TLS survival (Table 4). Furthermore, we investigated the value of immunophenotype, chromosome karyotype, gene fusion, and treatment duration in the prognosis of TLS, and found no significant association between event-free survival and clinical features as well as treatment duration (Fig. 3). Nevertheless, a poorer prognosis was observed in TLS children with abnormal karyotype and a hydration treatment of shorter than 7 days.

**Table 4:** Laboratory index in TLS patients with different outcome.

	Event-free survival (n = 71, %)	Relapse and death (n = 7, %)	OR (95% CI)
**WBC**^**a**^			
< 50 × 10^9^/L	46.5	28.6	1.00
≥ 50 × 10^9^/L	53.5	71.4	2.2 (0.4–11.9)
**Hyperkalemia**			
No	62.0	85.7	1.00
Yes	38.0	14.3	0.2 (0.0–2.4)
**Hyperphosphatemia**			
No	29.6	57.1	1.00
Yes	70.4	42.9	0.3 (0.1–1.5)
**Hyperuricemia**			
No	45.1	28.6	1.00
Yes	54.9	71.4	2.1 (0.4–11.3)
**Hypocalcemia**			
No	50.7	42.9	1.00
Yes	49.3	57.1	1.4 (0.3–6.6)
**Elevated creatinine**			
No	57.7	57.1	1.00
Yes	42.3	42.9	1.0 (0.2–4.9)

^a^Maximum number of WBC.

## Discussion

In the present study, we made a comprehensive analysis on the clinical features and risk factors of TLS. The incidence of TLS in our study was 1.4%, lower than previously reported 20%^7^. The difference may be attributed to the preventive measures in CCCG-ALL 2015 protocol, such as sufficient hydration and controlling biochemical indexes. In addition, the clinical data reported in CCCG were complete. Therefore, we believe that the incidence of 1.4% was accurate.

A panel of TLS experts has developed guidelines for risk identification of TLS in adults and children with malignant diseases^8^. They put forward that ALL patients were at intermediate risk or high risk of TLS, depending on WBC and lactate dehydrogenase (LDH) levels at diagnosis. Another evidence-based review also pointed out that elevated circulating tumor cells were a risk factor of TLS in ALL patients^9^. In the present study, we compared demographic characteristics of 79 ALL children with TLS and 5458 ALL children without TLS. As expected, our results showed that older age and being an infant were risk factors of TLS. This was consistent with a previous study listing the risk factor of TLS^8^. Similarly, Gopakumar et al. reported children with TLS were likely to be older than those without TLS, although the difference was not significant^11^. Furthermore, ALL patients with TLS had remarkably higher WBC count, which is also aligned with the results in previous studies^8,14^. The proportion of T-ALL children was higher in the TLS group, indicating T-ALL cells may exert a heavier tumor burden on leukemia cells. Although these findings have been well documented in the literature, they are still valuable considering the large sample size of our study.

We have made several interesting observations by dividing TLS patient into two subgroups based on their WBC count. We found a higher proportion of T-ALL children in the high WBC count group,consistent with the literature. We also found that the incidences of hyperphosphatemia and hypocalcemia were significantly higher in TLS patients with hyperleukocytosis, indicating its significant impact on the severity of TLS. This can be interpreted by the fact that more phosphorus is released from leukemia cells. It was also interesting that the WBC count moderate in some patients. The reason could be elucidated with more clinical data of low-WBC-count ALL children without TLS. In addition, our results were consistent with previous reports that TLS commonly occurred within the first 3 days after chemotherapy was initiated^8,15^.

We summarized the effectiveness of chemotherapy regimens. It should be noted that rasburicase may be an effective therapy for hyperuricemia. However, it was just approved to be used in TLS by the National Medical Products Administration (NMPA) of China at the end of 2018. Therefore, we did not collect the data on this drug. More clinical data on using urate oxidase are needed to enhance our findings.

The Venn diagram showed that hypocalcemia and hyperphosphatemia were almost concomitant, which was in line with metabolic characteristics. To identify the association between clinical symptoms and laboratory indexes, we analyzed the biochemical profiles of patients with/without arrhythmias, seizures, and renal injury. The results suggested that renal injury might be an important feature in the process of TLS. It should be noted that these data were collected in the absence of urate oxidase administration, which is now considered a standard treatment for high-risk patients.

We next divided the subjects into subgroups according to their demographic feature and the basic feature of their disease (i.e. immunophenotype, chromosome abnormality, and gene fusion). The results showed that the mean calcium level was lower in males than in females. Clinically, phosphorus and calcium are physiologically linked, and hypocalcemia is often secondary to hyperphosphatemia caused by tumor cell lysis^6,16^. Hyperphosphatemia may cause soft tissue calcification^17^, while severe hypocalcemia can lead to arrhythmia and convulsion. In our present data, male patients had a significantly lower calcium level than females, indicating the critical role of calcium in the TLS mechanism. However, no obvious disparity was found in phosphorus levels between subgroups.

Another characteristic of TLS was hyperkalemia. Hyperkalemia is considered a life-threatening consequence of TLS due to the possibility of causing cardiac arrhythmia and cardiac arrest^4^. Hyperuricemia is also a manifestation of metabolic disturbances of TLS. When leukemia cells are disrupted, intracellular substances such as purine and their metabolites, are released in the serum. Uric acid is an end product of purine derivatives^18^. In our present study, there was no notable difference in kalium and uric acid levels between subgroups, indicating a relatively concordant metabolic change of these chemicals in males and females, older and younger, as well as B-ALL and T-ALL TLS patients.

Darmon et al. reported that about 64% of TLS patients experienced acute kidney injury (AKI)^19^. In the condition of kidney failure, the clearance of phosphorus, potassium, and uric acid is curbed^20^. However, in our 79 subjects, only 41.8% (33 patients) confronted significantly elevated serum creatinine. This discrepancy is partly due to the difference in disease types (i.e. Darmon et al. recruited acute myeloid leukaemia (AML), acute lymphoblastic leukemia (ALL), and aggressive NHL). We found serum creatinine was significantly higher in older age, as well as in T-ALL patients, indicating that these populations are more susceptible to kidney injury. However, we found no association of lab indexes with chromosome abnormality as well as gene fusion, which should be verified in future studies.

Ronald et al. from Mayo Clinic have evaluated the outcomes in hospitalized patients with TLS, using the National Inpatient Sample (NIS)^5^. They identified a series of prognostic factors of TLS, including age, cancer type, etc. In our present study, of the seven patients encountering relapse or death, four patients died within 1–2 months after TLS onset. We found no significant association between major laboratory indexes and TLS survival, which is inconsistent with the previous finding that a high WBC count was related to a poor prognosis^21^. This discrepancy may be explained by the small number of cases with relapse and death, and more data should be collected to explore the effect of lab values on TLS outcomes.

We found no significant association between event-free survival and other clinical features, such as immunophenotype, karyotype, and treatment regimen. Nevertheless, we found that abnormal karyotype and hydration < 7 days might indicate a worse prognosis of TLS patients, indicating the mechanistic role of chromosome karyotype and the obvious effectiveness of hydration treatment. It should be noted that death within 1–2 months was more likely a direct consequence of TLS. However, only four deaths were reported in the present study. Further studies with more death cases should be conducted to analyze prognostic factors of TLS. We showed the survival analysis to provide a reference for other studies and provide some potential clues for clinical treatment.

It should be stated that, for some of the TLS patients, we only recorded their normal data for a certain laboratory index and did not track down all the definite abnormal data. Therefore, they may not have two abnormal laboratory indicators in our dataset. However, there was a definite medical record showingthat they had met diagnostic criteria of TLS and most of them have obvious TLS symptoms (arrhythmia or renal injury). In addition, all of those patients accepted the treatment of TLS and were categorized as TLS patients in our CCCG dataset. Therefore, their data were also incorporated into our analysis. Although limitations exist in our recruitment, the real-world nature of our data is of significant value.

## Conclusion

Older age (≥ 10 years), younger age (≤ 1 year), higher WBC count and T-ALL are risk factors of TLS. Hyperleukocytosis can increase the severity of TLS. Hyperphosphatemia is the most frequent laboratory abnormality. Renal injury may be an important feature in the progression of TLS. Our results provided strong evidence for understanding and managing TLS complicated with childhood ALL.

## Supplementary information

Supplementary Informations.

## Data Availability

All data generated or analyzed during this study are included in this published article and its supplementary files.

## References

[1] Hunger SP, Lu X, Devidas M (2012). Improved survival for children and adolescents with acute lymphoblastic leukemia between 1990 and 2005: a report from the children's oncology group. J. Clin. Oncol..

[2] Bhojwani D, Yang JJ, Pui CH (2015). Biology of childhood acute lymphoblastic leukemia. Pediatr. Clin. North Am..

[3] Oskarsson T, Soderhall S, Arvidson J (2016). Relapsed childhood acute lymphoblastic leukemia in the Nordic countries: prognostic factors, treatment and outcome. Haematologica.

[4] Burns RA, Topoz I, Reynolds SL (2014). Tumor lysis syndrome: risk factors, diagnosis, and management. Pediatr. Emerg. Care.

[5] Durani U, Shah ND, Go RS (2017). In-hospital outcomes of tumor lysis syndrome: a population-based study using the national inpatient sample. Oncologist.

[6] Criscuolo M, Fianchi L, Dragonetti G, Pagano L (2016). Tumor lysis syndrome: review of pathogenesis, risk factors and management of a medical emergency. Expert Rev. Hematol..

[7] Howard SC, Jones DP, Pui CH (2011). The tumor lysis syndrome. N. Engl. J. Med..

[8] Cairo MS, Coiffier B, Reiter A, Younes A, T.L.S.E. Panel (2010). Recommendations for the evaluation of risk and prophylaxis of tumour lysis syndrome (TLS) in adults and children with malignant diseases: an expert TLS panel consensus. Br. J. Haematol..

[9] Coiffier B, Altman A, Pui CH, Younes A, Cairo MS (2008). Guidelines for the management of pediatric and adult tumor lysis syndrome: an evidence-based review. J. Clin. Oncol..

[10] Saeed F, Ali MS, Ashraf MS, Vadsaria K, Siddiqui DE (2018). Tumour lysis syndrome in children with haematological cancers: experience at a tertiary care hospital in Karachi. J. Pak. Med. Assoc..

[11] Gopakumar KG, Seetharam S, Km JK (2018). Risk-based management strategy and outcomes of tumor lysis syndrome in children with leukemia/lymphoma: analysis from a resource-limited setting. Pediatr. Blood Cancer.

[12] Cai J, Yu J, Zhu X (2019). Treatment abandonment in childhood acute lymphoblastic leukaemia in China: a retrospective cohort study of the Chinese Children's Cancer Group. Arch. Dis. Child..

[13] Shen S, Chen X, Cai J (2020). Effect of dasatinib versus imatinib in the treatment of pediatric Philadelphia chromosome-positive acute lymphoblastic leukemia: a randomized clinical trial. JAMA Oncol..

[14] Rahmani B, Patel S, Seyam O (2019). Current understanding of tumor lysis syndrome. Hematol. Oncol..

[15] Cairo MS, Bishop M (2004). Tumour lysis syndrome: new therapeutic strategies and classification. Br. J. Haematol..

[16] Ahn YH, Kang HJ, Shin HY (2011). Tumour lysis syndrome in children: experience of last decade. Hematol. Oncol..

[17] Abdullah S, Diezi M, Sung L (2008). Sevelamer hydrochloride: a novel treatment of hyperphosphatemia associated with tumor lysis syndrome in children. Pediatr. Blood Cancer.

[18] Micho H, Mohammed Y, Hailu D, Genet S (2018). Evaluation and characterization of tumor lysis syndrome before and after chemotherapy among pediatric oncology patients in Tikur Anbessa specialized hospital, Addis Ababa, Ethiopia. BMC Hematol..

[19] Darmon M, Vincent F, Camous L (2013). Tumour lysis syndrome and acute kidney injury in high-risk haematology patients in the rasburicase era. A prospective multicentre study from the Groupe de Recherche en Reanimation Respiratoire et Onco-Hematologique. Br. J. Haematol..

[20] Wilson FP, Berns JS (2014). Tumor lysis syndrome: new challenges and recent advances. Adv. Chronic Kidney Dis..

[21] Giammarco S, Chiusolo P, Piccirillo N (2017). Hyperleukocytosis and leukostasis: management of a medical emergency. Expert Rev. Hematol..

