# Supplementary material for: Clinical characteristics of tumor lysis syndrome in childhood acute lymphoblastic leukemia
Source: Sci Rep. 2021 May 6;11:9656. doi: 10.1038/s41598-021-88912-2 (PMC8102476; doi:10.1038/s41598-021-88912-2)

Clinical Characteristics of Tumor lysis syndrome in childhood acute lymphoblastic leukemia

Yao Xue^1,2,†^, Jing Chen^3,†^, Siyuan Gao^1,2^, Xiaowen Zhai^5^, Ningling Wang^6^, Ju Gao^7^, Yu Lv^8^, Mengmeng Yin^9^, Yong Zhuang^10^, Hui Zhang^11^, Xiaofan Zhu^12^, Xuedong Wu^13^, Chi Kong Li^14^, Shaoyan Hu^15^, Changda Liang^16^, Runming Jin^17^, Hui Jiang^18^, Minghua Yang^19^, Lirong Sun^20^, Kaili Pan^21^, Jiaoyang Cai^3^, Jingyan Tang^3^, Xianmin Guan^4,^* , Yongjun Fang^1,2,^*

1 Department of Hematology and Oncology, Children's Hospital of Nanjing Medical University, Nanjing, China

2 Key Laboratory of Hematology, Nanjing Medical University, Nanjing, China

3 National Children’s Medical Center, Department of Hematology/Oncology, Key Laboratory of Pediatric Hematology and Oncology of China Ministry of Health, Shanghai Children’s Medical Center, Shanghai Jiao Tong University School of Medicine, Shanghai, China

4 Department of Hematology/Oncology; Ministry of Education Key Laboratory of Child Development and Disorders; National Clinical Research Center for Child Health and Disorders; China International Science and Technology Cooperation base of Child development and Critical Disorders; Chongqing Key Laboratory of Pediatrics; Children’s Hospital of Chongqing Medical University, Chongqing, China.

5 Hematology Department, Children's Hospital of Fudan University, Shanghai, China

6 Department of Pediatric, The Second Hospital of Anhui Medical University, Hefei, China

7 Department of Pediatrics, West China Second University Hospital, Sichuan University; Key Laboratory of Birth Defects and Related Disease of Women and Children, Ministry of Education, Chengdu, China

8 Department of Hematology, Kunming Children's Hospital, Kunming, China

9 Department of Pediatric hematology, Tongji Hospital of Tongji medical college, Huazhong University of Science and Technology, Wuhan, China

10 Department of Pediatrics, Qilu Hospital of Shandong University, Jinan, China

11 Department of Hematology & Oncology, Guangzhou Women and Children's Medical Center, Guangzhou, China

12 State Key Laboratory of Experimental Hematology and Division of Pediatric Blood Diseases Center, Institute of Hematology and Blood Diseases Hospital, Chinese Academy of Medical Sciences and Peking Union Medical College, Tianjin, China

13 Department of Pediatrics, Nanfang Hospital, Southern Medical University, Guangzhou, China

14 Department of Pediatrics, Hong Kong Children’s Hospital, The Chinese University of Hong Kong, Hong Kong SAR, China

15. Department of Hematology/Oncology, Children’s Hospital of Soochow University, Suzhou, China

16. Department of Hematology/Oncology, Jiangxi Provincial Children’s Hospital, Nanchang, China

17. Department of Pediatrics, Union Hospital of Tongji medical college, Huazhong University of Science and Technology, Wuhan, China

18. Department of Hematology/Oncology, Shanghai Children’s Hospital, Shanghai Jiao Tong University, Shanghai, China

19. Department of Pediatrics, Xiangya Hospital Central South University, Changsha, China

20. Department of Pediatrics, Affiliated Hospital of Qingdao University, Qingdao, China

21. Department of Hematology/Oncology, Northwest Women’s and Children’s Hospital, Xi’an, China

*Correspondence to: Yongjun Fang, Department of Hematology and Oncology, Children's Hospital of Nanjing Medical University; Key Laboratory of Hematology, Nanjing Medical University. No. 72 Guangzhou Road, Nanjing 210008, China. Tel: 86-25-83117508; Fax: 86-25-83304239; Email: fyj322@189.cn

Xianmin Guan, Department of hematology/oncology; Ministry of Education Key Laboratory of Child Development and Disorders; National Clinical Research Center for Child Health and Disorders; China International Science and Technology Cooperation base of Child development and Critical Disorders; Chongqing Key Laboratory of Pediatrics; Children’s Hospital of Chongqing Medical University, Chongqing, China. Tel: +86 18623101413; Email: 7221751@qq.com

Key words: Tumor lysis syndrome, childhood ALL, clinical characteristic, Chinese Children’s Cancer Group

Running Title: TLS in childhood ALL

Supp Table 1. Distribution of laboratory index in TLS patients stratified by population characteristics

|  | Gender | | *P* | Age | | *P* |
| --- | --- | --- | --- | --- | --- | --- |
|  | Male (n=56) | Female (n=23) |  | ＜1 (n=7) | ≥1 (n=72) |  |
| WBC^ab^ (×10^9/L) | 87.3  (22.6 ~335.4) | 43.1  (13.0 ~183.9 ) | 0.171 | 146.9  (67.3 ~497.6 ) | 73.1  (15.1 ~322.1 ) | 0.234 |
| P^b^ (mmol/L) | 2.8 ( 2.1~4.2) | 2.5 (1.7~4.2) | 0.376 | 2.9 (2.3~3.8) | 2.7 (1.9~4.2) | 0.697 |
| UA^b^ (umol/L) | 546.5 (336.4~922.0) | 504.7 (342.0~822.1) | 0.898 | 697.4 (310.9~912.9) | 516.0 (344.2~881.5) | 0.986 |
| K (mmol/L) | 5.3±1.4 | 4.9±1.3 | 0.153 | 5.6±1.1 | 5.2±1.4 | 0.385 |
| Ca (mmol/L) | 1.7±0.5 | 2.0±0.6 | **0.030** | 1.5±0.3 | 1.8±0.6 | 0.158 |
| Cr^b^ (mmol/L) | 69.6 (39.2~126.0) | 50.0 (32.0~77.0) | 0.093 | 43.0 (8.0~47.4) | 68.5 (38.0~119.0) | **0.018** |
| BUN^b^ (mmol/L) | 13.4 (10.0~25.5) | 13.8 (7.3~20.9) | 0.214 | 10.5 (9.5~12.9) | 13.9 (9.8~25.0) | 0.159 |
| ALT^b^ (U/L) | 40.0 (30.0~65.0) | 37.0 (22.0~67.5) | 0.921 | 52.5 (36.0~58.0) | 40.0 (25.9~67.5) | 0.618 |
| LDH^b^ (U/L) | 2136.0 (1026.0~4632.0) | 2462.0 (973.0~3824.2) | 0.805 | 4121.0 (2339.0~4632.0) | 2094.0 (962.0~3824.2) | 0.110 |
| PCT^b^ ((ng/ml) | 0.8 (0.2~1.7) | 0.4 (0.1~0.9) | 0.165 | 1.1 (0.8~1.2) | 0.4 ( 0.2~1.5) | 0.242 |
| FePro^b^ (ng/ml) | 282.8 (183.8~446.2） | 512.9 ( 190.2~666.5) | 0.085 | 261.5 (149.9~418.4) | 357.0 (188.6~585.0) | 0.401 |

^a^ Maximum number of WBC

^b^ represented by median (25 centile ~ 75 centile) because of non-normal distribution

K - potassium; Ca - calcium; P – phosphorus; UA - uric acid; Cr – creatinine; BUN – blood urea nitrogen; ALT – alamine aminotransferase; LDH – lactate dehydrogenase; PCT – procalcitonin; FePro - ferritin

Supp Table 2. Distribution of laboratory index in TLS patients stratified by clinical information

|  | Immunophenotype | | *P* | Chromosome | | *P* | Molecular | | *P* |
| --- | --- | --- | --- | --- | --- | --- | --- | --- | --- |
|  | T-ALL (n=37) | B-ALL (n=42) |  | Normal (n=49) | Abnormal (n=23) |  | Normal (n=49) | Abnormal (n=30) |  |
| WBC^ab^ (×10^9/L) | 230.7  (73.8~400.9 ) | 21.7  (10.5~125.5) | **<0.001** | 76.2  ( 17.1~302.7 ) | 70.0  (13.9~312.8) | 0.786 | 67.3  ( 18.4~271.8) | 134.4  (14.7 ~402.2) | 0.449 |
| P^b^ (mmol/L) | 3.5 (1.9~4.2) | 2.5 (2.0~3.6) | 0.193 | 2.7 (1.9~4.4) | 2.7 (2.1~3.9) | 0.896 | 3.1 (1.9~4.2) | 2.5 (2.0~3.6) | 0.400 |
| UA^b^ (umol/L) | 516.0 (306.7~796.3) | 570.0 (362.0~948.3) | 0.469 | 444.0 (342.0~710.7) | 570.0 (218.0~915.0) | 0.442 | 570.0 (347.0~928.0) | 474.0 (267.8~851.3) | 0.611 |
| K (mmol/L) | 5.3±1.5 | 5.1±1.2 | 0.690 | 5.1±1.3 | 5.4±1.4 | 0.473 | 5.4±1.4 | 4.9±1.3 | 0.101 |
| Ca (mmol/L) | 1.7±0.6 | 1.9±0.6 | 0.218 | 1.7±0.5 | 2.0±0.7 | 0.138 | 1.8±0.5 | 1.8±0.7 | 0.591 |
| Cr^b^ (mmol/L) | 79.0 (45.0~124.0) | 48.7 (32.8~72.3) | **0.032** | 61.0 (38.0~109.7) | 50.0 (32.0~128.0) | 0.717 | 70.5 (39.7~119.0) | 49.0 (33.5~94.3) | 0.186 |
| BUN^b^ (mmol/L) | 18.8 (10.0~27.4) | 12.4 (9.4~17.2) | 0.074 | 13.9 (9.7~24.6) | 12.6 (10.4~23.0) | 0.672 | 15.0 (10.4~24.9) | 11.5 (9.4~20.3) | 0.309 |
| ALT^b^ (U/L) | 45.5 (28.0~77.2) | 38.9 (25.4~61.3) | 0.551 | 39.5 (26.3~61.8) | 40.0 (24.0~82.0) | 0.589 | 47.5 (28.5~67.5) | 37.0 (24.9~58.1) | 0.511 |
| LDH^b^ (U/L) | 2942.0 (2054.5~7480.1) | 1508.0 (615.3~3126.8) | **0.002** | 2154.0 (1091.5~3824.2) | 2169.0 (1114.0~4632.0) | 0.835 | 2178.5 (1051.8~4363.4) | 1882.0 ( 716.2~3751.3) | 0.267 |
| PCT^b^ ((ng/ml) | 0.2 (0.1~0.9) | 0.9 (0.2~1.8) | **0.042** | 0.3 (0.2~1.7) | 0.9 ( 0.2~1.3) | 0.371 | 0.6 ( 0.2~1.7) | 0.3 (0.2~1.1) | 0.223 |
| FePro^b^ (ng/ml) | 315.6 (160.1~396.4) | 380.9 (207.0~664.2) | 0.070 | 267.4 (153.9~473.0) | 376.5 (221.6~637.1 ) | 0.075 | 262.7 (169.4~468.1) | 404.4 (191.8~701.3) | 0.052 |

^a^ Maximum number of WBC

^b^ represented by median (25 centile ~ 75 centile) because of non-normal distribution

K - potassium; Ca - calcium; P – phosphorus; UA - uric acid; Cr – creatinine; BUN – blood urea nitrogen; ALT – alamine aminotransferase; LDH – lactate dehydrogenase; PCT – procalcitonin; FePro - ferritin

Figure Legend

Supplementary Fig.1 Length of time of each treatment strategies, including hydration, diuresis and allopurinol.


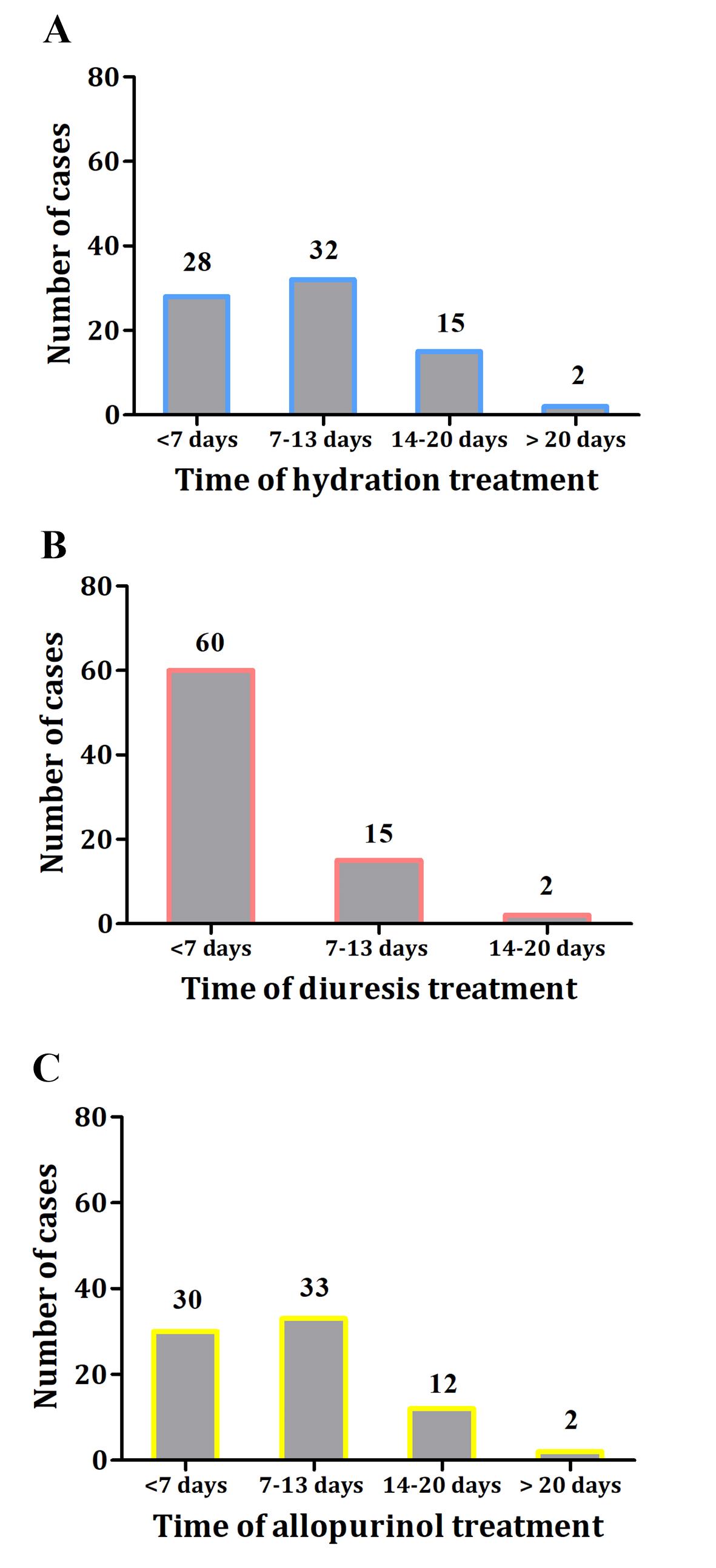

Supplement: Supplementary file 1 — Supplementary Informations. [file 41598_2021_88912_MOESM1_ESM.docx]
